# Effect of Quercetin Rich Onion Extracts on Bacterial Quorum Sensing

**DOI:** 10.3389/fmicb.2019.00867

**Published:** 2019-04-24

**Authors:** B. X. V. Quecan, J. T. C. Santos, M. L. C. Rivera, N. M. A. Hassimotto, F. A. Almeida, U. M. Pinto

**Affiliations:** ^1^Food Research Center (FoRC), Faculty of Pharmaceutical Sciences, University of São Paulo, São Paulo, Brazil; ^2^Department of Nutrition, Federal University of Juiz de Fora, Governador Valadares, Brazil

**Keywords:** quorum sensing, antimicrobial activity, onion, quorum quenching, phenolic compounds, glycosylation

## Abstract

Quorum sensing (QS) regulates bacterial gene expression and studies suggest quercetin, a flavonol found in onion, as a QS inhibitor. There are no studies showing the anti-QS activity of plants containing quercetin in its native glycosylated forms. This study aimed to evaluate the antimicrobial and anti-QS potential of organic extracts of onion varieties and its representative phenolic compounds quercetin aglycone and quercetin 3-β-D-glucoside in the QS model bacteria *Chromobacterium violaceum* ATCC 12472, *Pseudomonas aeruginosa* PAO1, and *Serratia marcescens* MG1. Three phenolic extracts were obtained: red onion extract in methanol acidified with 2.5% acetic acid (RO-1), white onion extract in methanol (WO-1) and white onion extract in methanol ammonium (WO-2). Quercetin 4-*O*-glucoside and quercetin 3,4-*O*-diglucoside were identified as the predominant compounds in both onion varieties using HPLC-DAD and LC-ESI-MS/MS. However, quercetin aglycone, cyanidin 3-*O*-glucoside and quercetin glycoside were identified only in RO-1. The three extracts showed minimum inhibitory concentration (MIC) values equal to or above 125 μg/ml of dried extract. Violacein production was significantly reduced by RO-1 and quercetin aglycone, but not by quercetin 3-β-D-glucoside. Motility in *P. aeruginosa* PAO1 was inhibited by RO-1, while WO-2 inhibited *S. marcescens* MG1 motility only in high concentration. Quercetin aglycone and quercetin 3-β-D-glucoside were effective at inhibiting motility in *P. aeruginosa* PAO1 and *S. marcescens* MG1. Surprisingly, biofilm formation was not affected by any extracts or the quercetins tested at sub-MIC concentrations. *In silico* studies suggested a better interaction and placement of quercetin aglycone in the structures of the CviR protein of *C. violaceum* ATCC 12472 than the glycosylated compound which corroborates the better inhibitory effect of the former over violacein production. On the other hand, the two quercetins were well placed in the AHLs binding pockets of the LasR protein of *P. aeruginosa* PAO1. Overall onion extracts and quercetin presented antimicrobial activity, and interference on QS regulated production of violacein and swarming motility.

## Introduction

Quorum sensing (QS) is a bacterial communication that uses signaling molecules known as autoinducers that accumulate in the medium according to population density (Fuqua et al., 1994; Whitehead et al., 2001; Lazdunski et al., 2004; Waters and Bassler, 2005). Signaling in Gram-positive microorganisms is mediated by low molecular weight peptides known as autoinducer peptides (AIPs) (Miller and Bassler, 2001). Other molecules such as autoinducer-2 (AI-2) are associated with most bacterial species allowing intra and interspecific communication (Miller and Bassler, 2001; Fuqua and Greenberg, 2002; Bai and Rai, 2011). Molecules such as quinolones, diketopiperazines and indole hydroxyketones can also function as communication cues (Waters and Bassler, 2005;Platt and Fuqua, 2010; Papenfort and Bassler, 2016).

In Gram-negative bacteria, signaling is usually mediated by acyl homoserine lactone (AHL) molecules, known as autoinducer-1 (AI-1) (Skandamis and Nychas, 2012). These molecules are composed of a fatty acid chain attached to a lactone ring by an amide bond. The variation that exists between the molecules of AHL occurs both by the size and the composition of the fatty acids that have a variation from 4 to 18 carbons and have some substitutions in the chain (Whitehead et al., 2001; Lazdunski et al., 2004; La Sarre and Federle, 2013). This mechanism was described in the 1970s in two species of bioluminescent marine bacteria: *Allivibrio fischeri* and *Vibrio harveyi* (Nealson and Hastings, 1979). In addition to these bacteria, there are other model microorganisms such as *Chromobacterium violaceum, Pseudomonas aeruginosa, Agrobacterium tumefaciens, Erwinia carotovora*, and *Serratia liquefaciens* in which QS has been well elucidated (Miller and Bassler, 2001; Waters and Bassler, 2005). There is great interest in these microorganisms as models to study QS, since many of the phenotypes are easily measured and are specifically regulated by QS.

In several bacteria QS regulates a range of phenotypes, coordinating a group behavior that controls the expression of virulence factors, extracellular enzymes, biofilm formation, secondary metabolites, motility, among others (Whitehead et al., 2001; Waters and Bassler, 2005; Skandamis and Nychas, 2012). Many of these phenotypes can impact food spoilage, making the product undesirable or unacceptable for consumption. As an example, the expression of some microbial extracellular enzymes like proteases, pectinases and lipases is regulated by QS (Ammor et al., 2008; Martins et al., 2018). Therefore, researchers have tried to find strategies to disrupt this communication using inhibitory compounds and consequently improve food quality and safety (Bai and Rai, 2011; Skandamis and Nychas, 2012).

Many studies have shown the potential of plant organic extracts rich in phenolic compounds to interfere with QS in different bacteria. These compounds constitute a diverse group of chemical substances, with different chemical activities, important for plant reproduction, growth, and protection against pathogens attack (Martínez et al., 2002). They can be classified depending on the ring number and the type of elements that bind them into phenolic acids, stilbenes, lignans, and flavonoids (Rodrigues et al., 2016).

The last group is an important class of natural products with polyphenol structure, widely found in fruits and vegetables (Panche et al., 2016). Its basic structural feature is the 2-phenyl-benzo-α-pyran compound which consists in two benzene rings (A and B) attached through a heterocyclic pyran ring (C) (Cushnie and Lamb, 2005). There is great interest in flavonoids because of their anti-inflammatory, antimicrobial, antioxidant and antitumor properties, among others (Cushnie and Lamb, 2005; Silveira, 2012; Rodrigues et al., 2016). In addition, flavonoids have also gained importance as potential inhibitors of the QS system. Different flavonoids such as taxifolin, kaempferol, naringenin, apigenin, baicalein, and others have demonstrated their ability to interfere in the QS system of microorganisms such as *P. aeruginosa* PAO1 and *C. violaceum* CV026 (Vandeputte et al., 2011), changing the transcription of QS-controlled target promoters and inhibiting the production of virulence factors (Paczkowski et al., 2017).

One of the most representative flavonoids found in high concentrations in foods, especially onion (284–486 mg/kg) is quercetin (Behling et al., 2008). Different studies showed the inhibitory potential of this compound against some microorganisms with phenotypes regulated by QS. A research performed by Gopu et al. (2015) evaluated the ability of quercetin against the QS biosensor strain *C. violaceum* CV026 and tested the anti-biofilm property of the compound against food-borne pathogens such as *Bacillus* spp., *Pseudomonas* spp. *Salmonella* spp., *Campylobacter jejuni*, and *Yersinia enterocolitica*. The results showed that quercetin inhibited violacein production in all the concentrations tested and additionally had a significant reduction of other phenotypes such as biofilm formation, exopolysaccharides, alginate production and motility in the compound’s presence (Gopu et al., 2015). Another study showed the effect of quercetin on biofilm formation and virulence factors’ production by *P. aeruginosa* PAO1 (Ouyang et al., 2016). The authors observed that quercetin had a significant inhibition on biofilm formation, pyocyanin, protease and elastase production. It was also observed that the expression of *lasI, lasR, rhII*, and *rhIR* genes was significantly reduced in response to quercetin (Ouyang et al., 2016).

Different types of quercetins such as quercetin aglycone, quercetin 4-glucoside, quercetin 3,4-*O*-diglucoside, quercetin 7,4-diglucoside, quercetin 3-glucosideglucoside and quercetin 5-glucoside are found in onion (*Allium cepa* Lineu). The anthocyanin cyanidin has also been identified in purple onion cultivars that give reddish or purple coloration to the bulbs (Lombard et al., 2005). The amount of quercetin in onions varies according to the color and type of bulb, being distributed mainly in the skins and outer rings (Arabbi et al., 2004; Lombard et al., 2005; Corzo Martínez et al., 2007; Kwak et al., 2017).

Studies have suggested that quercetin, a flavonol present in high concentrations in onion (*Allium cepa*), presents anti-QS properties against some Gram-negative microorganisms. However, there are no studies showing the anti-QS activity of plants containing quercetin in its native glycosylated forms. Thus, the objective of this work was to assess the potential presented by onion extracts to interfere with bacterial cell-to-cell communication.

## Materials and Methods

### Bacterial Strains and Culture Conditions

The microorganisms used in this work were *Chromobacterium violaceum* ATCC 12472 (30°C/24 h), *Pseudomonas aeruginosa* PAO1 (37°C/24 h), and *Serratia marcescens* MG1 (30°C/24 h). All cultures were grown in Luria Bertani (LB) agar or broth containing peptone 1%, yeast extract 0.5%, sodium chloride 0.5% with 1.2% agar, as needed.

### Preparation, Extraction, and Characterization of Phenolic Compounds of Onion Varieties

#### Preparation of Extracts

The extracts were prepared in the Laboratory of Chemistry, Biochemistry and Molecular Biology of Food in the Faculty of Pharmaceutical Sciences of the University of São Paulo. Samples of 5 kg of white and red onion (*Allium cepa*) were purchased from *Companhia de Entrepostos e Armazéns Gerais de São Paulo* (CEAGESP) warehouse. The samples were selected, cut and frozen with liquid nitrogen and stored at -80°C until use. For the analysis, 20 g of each onion variety were homogenized for 1 min using Ultra-Turrax (Polytron-Kinematica GmbH, Kriens-Luzern, Switzerland) in 100 ml of 70% methanol for white onion and 70% methanol acidified with 5% acetic acid for red onion due to its content of anthocyanins. Then, the samples were vacuum filtered, and the residue was recovered, repeating the process twice using 50 ml of the respective solvent. The obtained extracts were pooled and concentrated in a rotary evaporator (Rotavapor 120, Büchi, Flawil, Switzerland) at a temperature of 40°C until complete methanol removal, in order to use it for the solid phase separation step.

#### Solid Phase Extraction

Methanol free samples were loaded in a column with 1 g of polyamide (CC 6, Macherey-Nagel, Germany), prepared in a syringe of 6 ml and preconditioned passing 20 ml of methanol and 60 ml of distilled water. After application of the white onion extract, the column was washed with 20 ml of water and the elution of the flavonoids was performed with 50 ml of methanol and 50 ml of methanol: ammonium (95.5: 0.5 v/v), named WO-1 and WO-2 extracts, respectively. For red onion, the elution of the flavonoids was performed with 50 ml methanol acidified with 2.5% acetic acid, naming the extract as RO-1. The obtained eluates were completely dried in a rotary evaporator at 40°C and suspended in 1 ml of methanol. These extracts were used for the identification and quantification of total phenolic compounds using high-performance liquid chromatography with diode array detector (HPLC-DAD) and liquid chromatography-electrospray ionization-tandem mass spectrometry (LC-ESI-MS/MS).

#### High-Performance Liquid Chromatography With Diode Array Detector (HPLC-DAD)

Quantification and partial identification of flavonoids were conducted using HPLC-DAD. The chromatograph (Infinity 1120 model, Agilent, Germany) used was equipped with automatic sample injector, quaternary pump and DAD, controlled by Agilent’s own software. The column used was Prodigy 5 (ODS3 250 × 4.60 mm, Phenomenex Ltd., United Kingdom) with a flow rate of 1 ml/min, 25°C. The elution was performed with a solvent gradient with the following elements: A: water with 0.5% formic acid; B: Acetonitrile with 0.5% formic acid. The concentration gradient of the solvents was made with 8% of B at the beginning, 10% in 5 min, 17% in 10 min, 25% in 15 min, 50% in 25 min, 90% in 30 min, 50% in 32 min, and 8% in 35 min (running time, 35 min). The run was monitored with the following wavelengths: 270, 370, and 525 nm and peak identification was performed comparing the retention time and similarity with the absorption spectra of commercial patterns and the spectra contained in the equipment library, previously inserted in the method. The identification was also performed according to the sequence of elution according to Pérez Gregorio et al. (2011). For the quantification the following flavonoid standards were used: quercetin 3-*O*-glucoside, isorhamnetin and cyanidin 3-*O*-glucoside (Extrasynthese, Genay, France). All quercetin derivates were quantified and values expressed as quercetin 3-*O*-glucoside. All isorhamnetin derivates were quantified and values expressed as isorhamnetin equivalent. Cyanidin 3-*O*-glucoside was quantified, and value expressed as cyanidin 3-*O*-glucoside.

#### Liquid Chromatography-Electrospray Ionization-Tandem Mass Spectrometry (LC-ESI-MS/MS)

The identification of flavonoids and other phenolic compounds was conducted in the liquid chromatography (LC) (Prominence model, Shimadzu, Japan) linked to a mass spectrometer ion trap (Esquire HCT model, Bruker Daltonics, Germany) and electrospray ionization interface (ESI). The separation conditions were the same as those used for the HPLC-DAD, described in section High-Performance Liquid Chromatography With Diode Array Detector (HPLC-DAD). After passage through the DAD, the flow was changed to 0.2 ml/min to the passage in the mass spectrometer. The ESI was maintained in positive mode. The mass detector was programmed to perform full scan between *m/z* 100–1000. The ionization energy for the positive mode was 3500 V. The identity of the compounds was evaluated by comparing the mass spectrum obtained with the commercial standards and, or literature data (Lee and Mitchell, 2011). To confirm the identity, the HPLC retention time of commercial flavonoid standards (quercetin 3-*O*-glucoside, quercetin aglycone, and cyanidin 3-*O*-glucoside) was used for comparison.

### Antimicrobial Activity of the Extracts and Isolated Compounds of Onions

#### Minimal Inhibitory Concentration of the Extracts

The minimal inhibitory concentration (MIC) of each extract was determined using the broth microdilution method, according to the methodology of Wiegand et al. (2008), with modifications. The extracts suspended in LB broth were tested in a 96-well plate. Cultures of *C. violaceum* ATCC 12472, *P. aeruginosa* PAO1, and *S. marcescens* MG1 were grown overnight on plates with LB agar, suspended in saline solution 0.85% and adjusted using a solution of McFarland 0.5 to reach a concentration of approximately 1 × 10^8^ CFU/ml. Subsequently, each culture was diluted in LB broth in a proportion of 1:100 and 50 μl of this dilution were placed in each well, to attain the final concentration ranging from 31.2 to 125 μg/ml of extract. The controls were bacterial culture in LB broth without extracts, the broth with each of the extracts in each of the concentrations tested without bacteria, and a sterility control. The QS inhibition tests were prepared with sub-MIC concentrations to ensure that the extracts did not interfere with bacterial growth. Bacterial growth was evaluated following the same procedure for the MIC. Optical density at 595 nm (OD 595 nm) was determined each 3 h during a total time of 24 h using the spectrophotometer (Multiskan FC, Thermo Fisher Scientific, Finland). Quercetin aglycone and quercetin 3-β-D-glucoside were also evaluated as onion’s representative isolated compounds.

### Quorum Sensing Modulation Assays by Extracts and Isolated Compounds of Onions

#### Violacein Production in *C. violaceum* ATCC 12472

The test was performed according to Tan et al. (2012, 2013), with modifications. *C. violaceum* ATCC 12472 was grown overnight following the same parameters as in the section Minimal Inhibitory Concentration of the Extracts. In concentrations ranging from 7.8 to 31.2 μg/ml. A 96-well plate was incubated at 30°C, 120 rpm for 24 h and then the plates were completely dried at 60°C. Subsequently, 100 μl of dimethyl sulfoxide (DMSO) were added to each well, keeping the plate with agitation at 120 rpm for 12 h, approximately. The OD 595 nm was measured using the spectrophotometer (Multiskan FC, Thermo Fisher Scientific, Finland). The controls used in the test were the same as in the section Minimal Inhibitory Concentration of the Extracts. Quercetin aglycone and quercetin 3-β-D-glucoside were also evaluated for their anti-QS activity for being representative isolated compounds found in the extracts.

#### Swarming Motility by *P. aeruginosa* PAO1 and *S. marcescens* MG1

Swarming motility was tested using semi-solid LB medium prepared with 0.5% agar, as described by Oliveira et al. (2016). Aliquots of the extracts giving final concentrations of 31.2, 62.5, and 125 μg/ml were placed in sterile Petri dishes of 49 × 9 mm and then 10 ml of the molten agar were added. For the swarming test 2 μl of the overnight grown bacteria were point inoculated at the center of the agar. Once the inoculum was dried, about 20 min after inoculation, the plates were closed and incubated at 37°C for 24 h for *P. aeruginosa* PAO1 and at 30°C for 24 h for *S. marcescens* MG1. Inhibition of swarming motility was considered when a visual reduction of the swarm was observed in presence of the extracts. Quercetin aglycone and quercetin 3-β-D-glucoside were also evaluated for their anti-QS activity. Synthetic furanone C-30 (≥97.0% of purity; Sigma-Aldrich, Brazil) (Z-)-4-Bromo-5-(bromomethylene)-2(5H)-furanone, was used as positive control for motility inhibition at 100 μM (Oliveira et al., 2016).

#### Biofilm Formation in *P. aeruginosa* PAO1 and *S. marcescens* MG1

The effect of onion extracts on biofilm formation was assessed in a 96-well plate as it was described by Borges et al. (2012), with modifications. An aliquot of 20 μl of the overnight cultures adjusted according to McFarland solution 0.5 were inoculated into LB broth with 31.2, 62.5, and 125 μg/ml of extract, completing a final volume of 200 μl. The cultures were incubated at 37°C for 24 h when using *P. aeruginosa* PAO1 and at 30°C for 24 h when evaluating *S. marcescens* MG1. Thereafter, non-adherent bacteria were removed by washing with 200 μl of saline solution 0.85% and adherent bacteria were fixed with 200 μl of methanol 99% for 15 min, following removal of the solvent. Then 200 μl of crystal violet solution 0.3% (w/v) were added to the well for 5 min. The wells were washed with sterile water to remove excess stain and the crystal violet bound to the biofilm was extracted with glacial acetic acid 33% (v/v). The OD 595 nm of the crystal violet solution was measured using the spectrophotometer (Multiskan FC, Thermo Fisher Scientific, Finland). Quercetin aglycone and quercetin 3-β-D-glucoside were also evaluated.

To confirm biofilm production in the case of the microorganism *P. aeruginosa* PAO1, a test was performed according to Minei et al. (2008) with modifications. The 96-well plates with cultures grown overnight in LB broth and the different compounds to be tested were incubated, as previously mentioned. Planktonic cells were removed with 200 μl of sterile saline solution 0.85%. Shortly, the adhered cells and biofilm were removed manually scrubbing the walls of each well with a sterile swab until the biofilm was completely removed, and then the swab was transferred to a tube containing 10 ml of saline solution 0.85% and vortexed for 1 min. Serial dilutions were made, and an inoculum of 20 μl was plated using the drop plate method in LB agar following incubation at 37°C monitoring the plates until the appearance of the micro-colonies. After that, cells were counted, and the results were expressed as Log_10_ CFU/biofilm formed into the well.

### Molecular Docking of Quercetin Molecules With CviR and LasR Proteins

Docking studies were performed according to Almeida et al. (2016) and Almeida et al. (2018). In brief, the crystallized structures of CviR protein of *C. violaceum* ATCC 12472 (PDB: 3QP6 and 3QP8; Chen et al., 2011) and LasR protein of *P. aeruginosa* PAO1 (PDB: 2UV0, 6D6A, 6D6L, 6D6O, and 6D6P; Bottomley et al., 2007; O’Reilly et al., 2018) with different ligands were obtained in the RCSB Protein Data Bank database (PDB)^1^. Then, the molecular docking was performed between these proteins and *N*-(3-hydroxydecanoyl)-DL-homoserine lactone (3-OH-C10-HSL; Pubchem CID: 71353010), *N*-(3-oxododecanoyl)-L-homoserine lactone (3-oxo-C12-HSL; Pubchem CID: 3246941), quercetin (quercetin aglycone; Pubchem CID: 5280343), quercetin 3,4-*O*-diglucoside (quercetin 3-β-D-glucoside; Pubchem CID: 5280804) and 4-bromo-5-(bromomethylene)-2(5H)-furanone (Furanone C-30; Pubchem CID: 10131246) using the “Dock Ligands” tool of the CLC Drug Discovery Workbench 4.0 software^2^, with 1000 interactions for each compound and the conformation of the compounds was changed during the docking via rotation around flexible bonds. The generated score mimics the potential energy change when the protein and the compound come together based on hydrogen bonds, metal ions and steric interactions, where lower scores (more negative) correspond to higher binding affinities. The five best scores of the docking of each compound were selected, allowing the inspection of the binding sites of CviR and LasR proteins with each compound (Almeida et al., 2016, 2018).

### Statistical Analysis

All experiments were performed at least three times. The data represent the means of the repetitions and their differences with respect to the controls. All data were subjected to analysis of variance (ANOVA) followed by Tukey’s test using the Statistical Analysis System and Genetics Software (Ferreira, 2011). A *p* < 0.05 was considered to be statistically significant.

## Results

### Characterization of the Phenolic Compounds Present in Onion Samples

**FIGURE 1:**
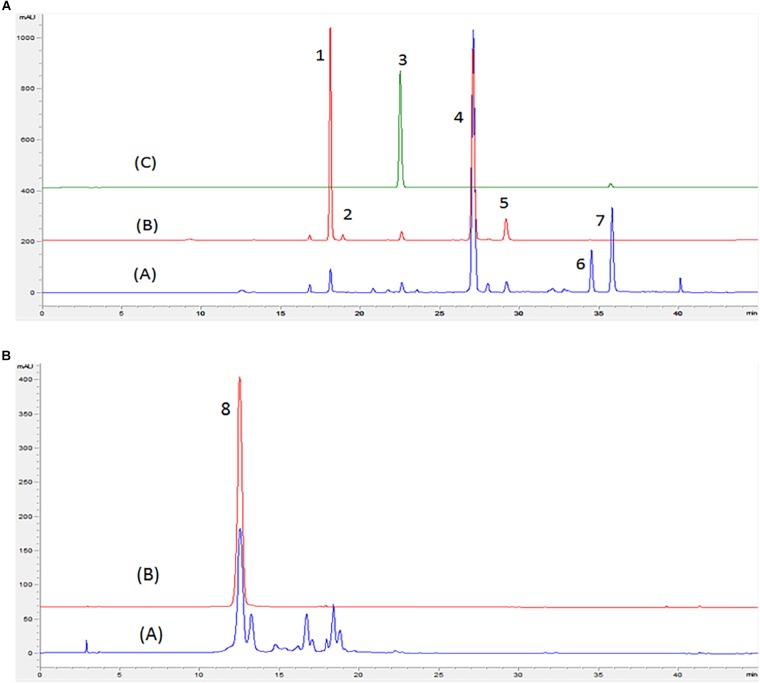
Chromatogram obtained by HPLC-DAD in wavelengths of 370 **(A)** and 525 nm **(B)** of red and white onion. (A) Red onion, (B) White onion, (C) Quercetin 3-*O*-glucoside standard. Peaks identified: peak 1 – Quercetin 3,4-*O*-diglucoside; peak 2 – Isorhamnetin 3,4′-diglucoside, peak 3 – Quercetin 3-*O*-glucoside; peak 4 – Quercetin 4′-*O*-glucoside; peak 5 – Isorhamnetin 4- glucoside; peak 6 – Quercetin glycoside; peak 7 – Quercetin aglycone; peak 8 –Cyanidin 3-*O* glucoside. Identification shown in Table 1.

Chromatograms obtained by HPLC-DAD of flavonoids from red and white onion extracts are shown in Figure 1 and their respective identification detailed in Table 1. The LC-ESI-MS/MS spectra of chromatographic peaks obtained from red and white onions are also shown in Supplementary Figure S1. The major flavonol identified in the two onion varieties was quercetin 4-*O*-glucoside. The second major flavonol found was identified as quercetin 3,4-*O*-diglucoside. In addition, an anthocyanin in RO-1 extract was found. Cyanidin 3-*O*-glucoside, responsible for purple pigmentation of red onion, was presented with molecular ion [M]+ at *m/z* 449 and characteristic MS2 fragment at m/z 287 ([M]^+^ – 162). Concentrations of flavonoids from red and white onion extracts are shown in Table 2. Quercetin 4-*O*-glucoside and quercetin 3,4-*O*-diglucoside corresponded to 52 and 37% of total flavonoids in the WO-1 extract. The WO-2 extract was primarily composed of quercetin 4-*O*-glucoside. On the other hand, in RO-1 extract, the main flavonoids were quercetin 4-*O*-glucoside and quercetin aglycone, representing 57 and 20% of total flavonoids.

**Table 1:** Mass spectra of flavonoids in positive mode from red and white onion extracts obtained by LC-ESI-MS/MS.

Peak	RT (min)	Molecular ion (*m/z*)	MS2 (*m/z*)	Flavonoids	Red onion	White onion
1	18.1	627	465/303	Quercetin 3,4-*O*-diglucoside	✓	✓
2	19.0	641	479/317	Isorhamnetin3,4′-diglucoside		✓
3	22.7	465	303	Quercetin 3-*O*-glucoside^∗^	✓	✓
4	27	465	303	Quercetin 4′-*O*-glucoside	✓	✓
5	29.5	479	317	Isorhamnetin4-glucoside	✓	✓
6	34.4	507	303	Quercetin glycoside	✓	
7	35.2	303	257/229/165/137	Quercetin aglycone^∗^	✓	
8	11.9	449	287	Cyanidin 3-*O*-glucoside^∗^	✓	


*RT, retention time. ^∗^Identity confirmed with commercial standard.*

**Table 2:** Flavonoids content in red and white onion extracts.

	Red onion	White onion	
		
	RO-1	WO-1	WO-2
**FLAVONOL**			
**Quercetin 3,4-*O*-diglucoside**	0.684 ± 0.001	**7.846 ± 0.080**	–
Isorhamnetin3,4-diglucoside	–	0.191 ± 0.001	–
Quercetin 3-*O*-glucoside	0.359 ± 0.001	0.413 ± 0.001	–
**Quercetin 4-*O*-glucoside**	**10.546 ± 0.020**	**11.032 ± 0.010**	**1.478 ± 0.005**
Isorhamnetin4-glucoside	0.500 ± 0.001	1.263 ± 0.008	–
Quercetin glycoside	1.44 ± 0.004	–	–
**Quercetin aglycone**	**3.741 ± 0.050**	–	–
Total flavonol	17.272	20.738	1.478
**ANTHOCYANIN**			
**Cyanidin 3-*O*-glucoside**	**1.162 ± 0.007**	–	–


*–, not detected; RO-1, red onion extract in methanol acidified with 2.5% acetic acid; WO-1, white onion extract in methanol; WO-2, white onion extract in methanol ammonium. All quercetin derivates were expressed as equivalents of quercetin 3-O-glucoside. All isorhamnetin derivates were expressed as isorhamnetin. All results were expressed as mg/100 mg of dry extract. In bold, the major compounds identified.*

### Determination of Minimum Inhibitory Concentration (MIC) and Microbial Growth Curves in the Presence of Extracts and Isolated Compounds of Onions

The MIC results of red and white onion extract of *C. violaceum* ATCC 12472, *P. aeruginosa* PAO1, and *S. marcescens* MG1 are presented in Table 3. For QS inhibition experiments we used sub-MIC concentrations that did not affect microbial growth, according to growth curves. For *C. violaceum* ATCC 12472 both the RO-1 and the WO-2 extract had an inhibitory effect in a concentration of 125 μg/ml. In addition, bacterial multiplication was slightly affected in relation to the control for the two types of onion extracts in the concentration of 62 μg/ml, showing a partial inhibition of the growth. Thus, QS inhibition experiments were performed using concentrations below 62 μg/ml to avoid toxic effects. In the case of *P. aeruginosa* PAO1 bacteria grew, similarly, to the control in almost all extract concentrations tested. Only the WO-2 extract had a delayed exponential phase, compared to the control at 125 μg/ml. A similar trend was observed for *S. marcescens* MG1.

**Table 3:** Minimum inhibitory concentration of onion extracts.

Microorganism	MIC (μg/ml)				
	
	RO-1	WO-1	WO-2	Quercetin aglycone	Quercetin 3-β-D-glucoside
*C. violaceum* ATCC 12472	125	>125	125	>125	125
*P. aeruginosa* PAO1	>125	>125	>125	>125	>125
*S. marcescens* MG1	125	>125	>125	>125	>125


*RO-1, red onion extract in methanol acidified with 2.5% acetic acid; WO-1, white onion extract in methanol; WO-2, white onion extract in methanol ammonium.*

Growth curves were also performed in order to check the effect of quercetin aglycone and quercetin 3-β-D-glucoside on the microorganisms used in this study (Table 3). These compounds were chosen because the first one was found in the literature as a potential QS inhibitor besides being present in the RO-1 extract and the second as a representative compound of the glycosylated forms of quercetin found in both types of onion in the present study. The MIC for the two compounds was greater than 125 μg/ml. Additionally, for *C. violaceum* ATCC 12472 a partial inhibition of growth was observed at the concentration of 125 μg/ml of quercetin 3-β-D-glucoside, therefore we used lower concentrations to avoid toxic effects.

### Determination of Anti-QS Activity of Extracts and Isolated Compounds of Onions

#### Effect on Violacein Production in *C. violaceum* ATCC 12472

**FIGURE 2:**
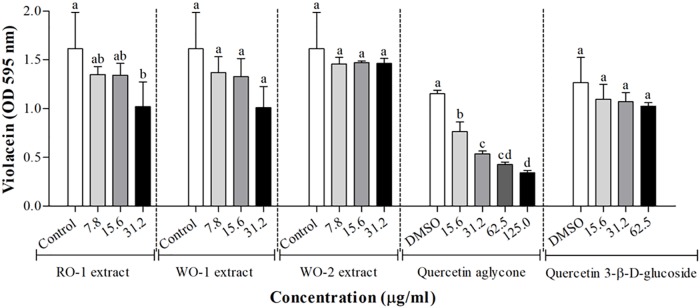
Violacein production in *C. violaceum* ATCC 12472 in the presence of RO-1 (Red onion extract in methanol acidified with 2.5% acetic acid), WO-1 (White onion extract in methanol), WO-2 (White onion extract in methanol ammonium), quercetin aglycone and quercetin 3-β-D-glucoside. Control, bacterial growth in LB; DMSO, bacterial control in LB plus DMSO; Means followed by different letters differ statistically (*p* < 0.05).

Figure 2 shows the effect of white and red onion extracts on violacein production in *C. violaceum* ATCC 12472. The production of violacein was statistically inhibited in the presence of 31.2 μg/ml of RO-1 extract when compared to the control (*p* < 0.05). On the other hand, WO-1 and WO-2 extracts did not influence the production of violacein, even though WO-1 presented an inhibitory tendency. It is noteworthy that only the RO-1 extract contains quercetin aglycone (Table 2).

As quercetin aglycone and quercetin 3-β-D-glucoside were molecules identified in the extracts and as the aglycone form has been reported as a potential QS inhibitor, the effect of onion extracts was compared to the effect of these two molecules Figure 2. For the aglycone form, the results showed that there was a significant inhibition of violacein production (*p* < 0.05). In contrast, quercetin 3-β-D-glucoside showed no significant inhibition of pigment production, even though a tendency can be observed, possibly explaining the low anti-QS activity of the extracts that had glycosylated forms of quercetin as major compounds.

#### Effect on Swarming Motility of *P. aeruginosa* PAO1 and *S. marcescens* MG1

**FIGURE 3:**
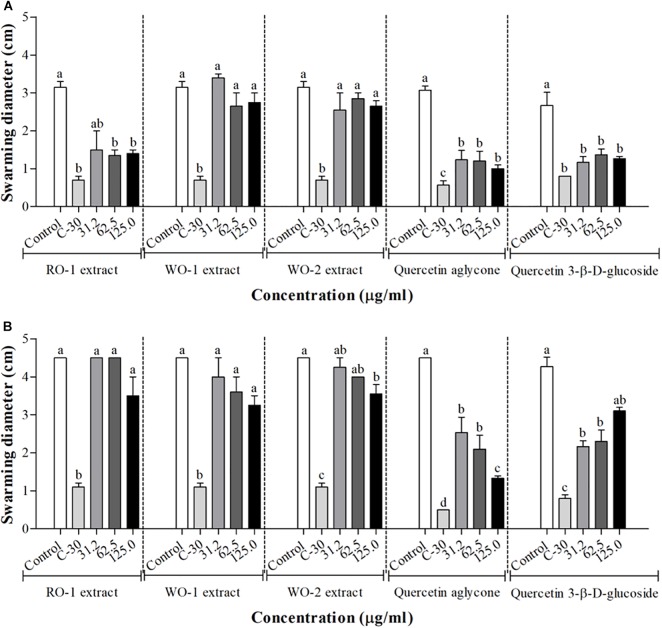
Swarming motility in *P. aeruginosa* PAO1 **(A)** and *S. marcescens* MG1 **(B)** in the presence of RO-1 (Red onion extract in methanol acidified with 2.5% acetic acid), WO-1 (White onion extract in methanol), WO-2 (White onion extract in methanol ammonium), quercetin aglycone and quercetin 3-β-D-glucoside. Control, bacterial growth in LB; C-30, Furanone C-30 with bacterium; Means followed by different letters differ statistically (*p* < 0.05).

The results of the effect of onion extracts on swarming motility of *P. aeruginosa* PAO1 are shown in Figure 3A. The RO-1 extract significantly reduced motility in the tested concentrations, and the control with furanone C-30 demonstrated the best phenotype inhibition. The other extracts did not present significant inhibition in this assay.

For *S. marcescens* MG1, inhibition of swarming motility was clearly observed at the concentration of 125 μg/ml of the WO-2 extract Figure 3B. The other extracts showed no significant inhibition, despite a trend observed in higher concentrations. Additionally, our assays with quercetin aglycone and quercetin 3-β-D-glucoside revealed a significant inhibition of swarming motility in both bacteria (*p* < 0.05) (Figure 3A,B). The violacein production tests showed that quercetin aglycone had better inhibitory activity than the glycosylated quercetin in *C. violaceum* ATCC12472. However, the results from the swarming motility assay showed that both types of quercetin were able to inhibit bacterial motility on agar plates.

#### Effect on Biofilm Formation of *P. aeruginosa* PAO1 and *S. marcescens* MG1

**FIGURE 4:**
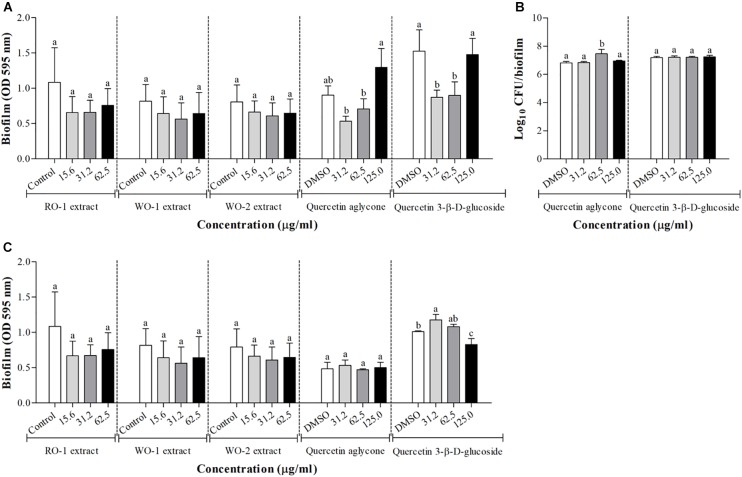
Biofilm formation in *P. aeruginosa* PAO1 **(A)** and *S. marcescens* MG1 **(C)** in the presence of RO-1 (Red onion extract in methanol acidified with 2.5% acetic acid), WO-1 (White onion extract in methanol), WO-2 (White onion extract in methanol ammonium), quercetin aglycone and quercetin 3-β-D-glucoside. DMSO, bacterial control in LB plus DMSO; Means followed by different letters differ statistically (*p* < 0.05); **(B)** Surface-adhered cell and cell in biofilm count after 24 h of incubation of *P. aeruginosa* PAO1 in the presence of quercetin aglycone and quercetin 3-β-D-glucoside.

Biofilm production was not significantly inhibited by any of the extracts as shown in Figure 4A for *P. aeruginosa* PAO1 and Figure 4C for *S. marcescens* MG1. Tests with quercetin aglycone and quercetin 3-β-D-glucoside in *P. aeruginosa* PAO1 showed inhibition at some concentrations, but paradoxically no inhibition was observed in the highest concentration tested. As this result was somewhat contradictory, we decided to use an additional technique to measure biofilm formation by counting viable cells recovered from the biofilms. However, the results of these counts did not reveal any inhibition of biofilm formation in any of the tested concentrations (Figure 4B), meaning that there was no difference in the counts of viable cells recovered from the biofilms at different concentrations of both types of quercetins. Figure 4C, shows the results of biofilm formation of *S. marcescens* MG1, revealing little to no inhibition by the tested molecules.

#### Molecular Docking of Quercetin Molecules With CviR and LasR Protein

**FIGURE 5:**
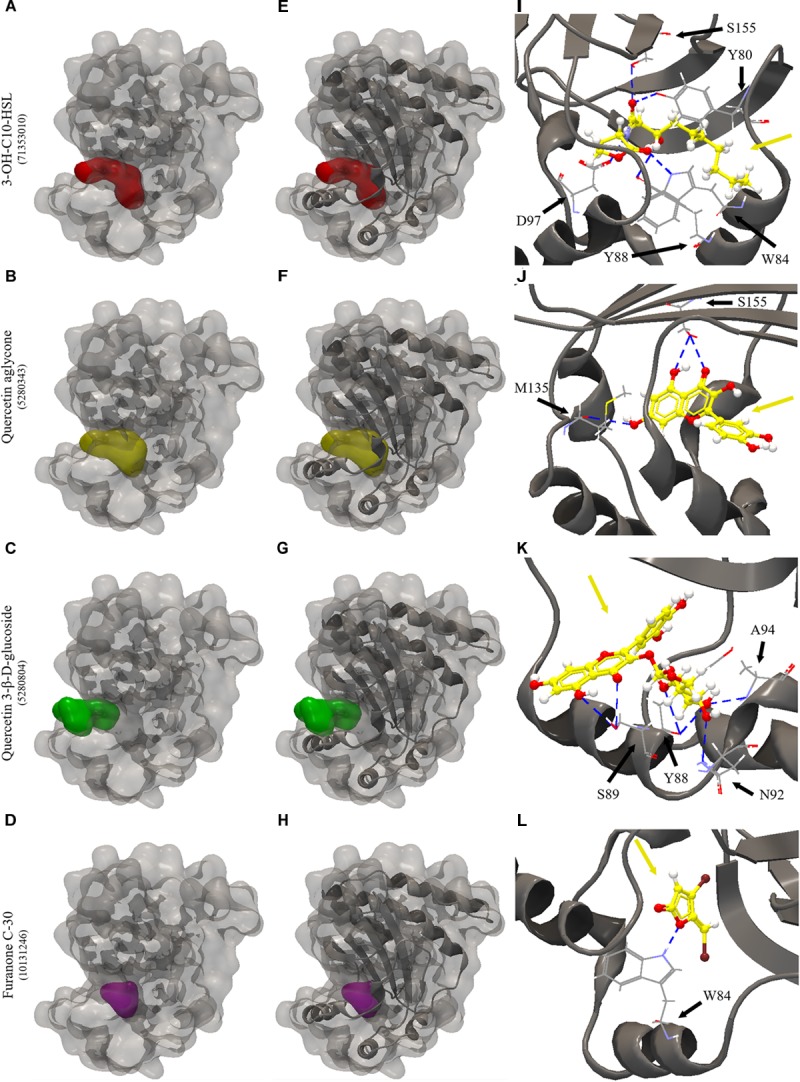
Molecular docking of 3QP8 structure of CviR protein of *C. violaceum* ATCC 12472 with 3-OH-C10-HSL, quercetin aglycone, quercetin 3-β-D-glucoside and furanone C-30. **(A–D)** surface representation of 3QP8 structure of CviR protein of *C. violaceum* ATCC 12472, **(E–H)** surface and backbone representations and **(I–L)** backbone representation with hydrogen bond between the amino acid residues and compounds evaluated. Gray surface representation, CviR protein; Red surface representation, 3-OH-C10-HSL; Yellow surface representation, quercetin aglycone; Green surface representation, quercetin 3-β-D-glucoside; Purple surface representation, furanone C-30; Gray backbone representation, CviR protein; Black arrow indicates the binding site; Yellow arrow, 3-OH-C10-HSL or quercetin aglycone or quercetin 3-β-D-glucoside or furanone C-30; Blue dashed line, hydrogen bond.

**FIGURE 6:**
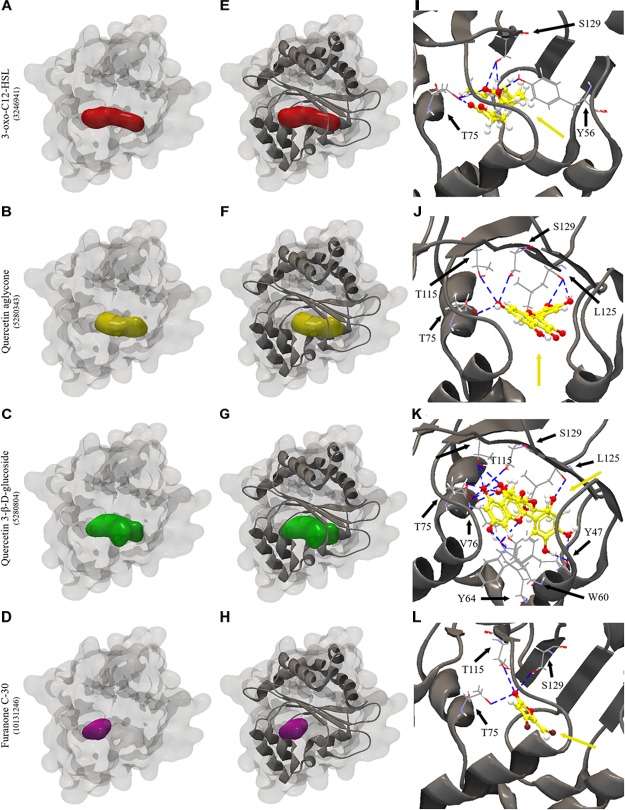
Molecular docking of 2UV0 structure of LasR protein of *P. aeruginosa* PAO1 with 3-oxo-C12-HSL, quercetin aglycone, quercetin 3-β-D-glucoside and furanone C-30. **(A–D)** surface representation of 2UV0 structure of LasR protein of *P. aeruginosa* PAO1, **(E–H)** surface and backbone representations and **(I–L)** backbone representation with hydrogen bond between the amino acid residues and compounds evaluated. Gray surface representation, LasR protein; Red surface representation, 3-oxo-C12-HSL; Yellow surface representation, quercetin aglycone; Green surface representation, quercetin 3-β-D-glucoside; Purple surface representation, furanone C-30; Gray backbone representation, LasR protein; Black arrow indicates the binding site; Yellow arrow, 3-oxo-C12-HSL or quercetin aglycone or quercetin 3-β-D-glucoside or furanone C-30; Blue dashed line, hydrogen bond.

All the evaluated compounds were able to bind in the evaluated structures of the CviR and LasR proteins and the binding affinities and binding residues are shown in Tables 4, 5. The 3-OH-C10-HSL presented the highest binding affinities for the two structures of CviR protein of *C. violaceum* ATCC 12472, 3QP6, and 3QP8. The quercetin aglycone presented lower binding affinities than this AHL and higher than quercetin 3-β-D-glucoside and furanone C-30 (Table 4). The quercetin aglycone bound to M135 and S155 residues from the two structures of the CviR protein evaluated, differently from quercetin 3-β-D-glucoside that bound at different sites (Table 4 and Figure 5). In addition, the S155 residue was a common binding site for quercetin aglycone and 3-OH-C10-HSL in these structures (Table 4 and Figure 5A,B). In 3QP8 structure, the flavonoid structure of quercetin 3-β-D-glucoside bound in the S89 residue and glucoside structure in residue Y88, N92, and A94 (Table 4). On the other hand, the different structures of the LasR protein of *P. aeruginosa* PAO1 showed variations of the binding affinities for the four evaluated compounds. The 3-oxo-C12-HSL showed the highest binding affinities for the structures 2UV0 and 6D6A and the quercetin 3-β-D-glucoside the highest binding affinities for the structures 6D6L, 6D6O, and 6D6P (Table 5). The T75, T115, and S129 residues were common binding sites for the two quercetins, 3-oxo-C12-HSL and furanone C-30 (Table 5 and Figure 6). These residues were also common binding sites for flavonoid structure of quercetin 3-β-D-glucoside (Table 5). On the other hand, the glucoside structure of this quercetin was able to bind in specific amino acid residues, such as G38, Y47, Y64, V76, L125, and A127 (Table 5). However, the inspection of the binding sites of CviR and LasR protein with these compounds showed that quercetin 3-β-D-glucoside was unable to accommodate in the pocket of the two structures of CviR protein of *C. violaceum*ATCC 12472 (Figure 5, 6).

**Table 4:** Results from molecular docking of structures of CviR protein of *C. violaceum* ATCC 12472 with selected compounds.

Compound	Pubchem CID	Structures of CviR protein of *C. violaceum* ATCC 12472					
		
		3QP6			3QP8		
			
		Binding residue	Score	Rank	Binding residue	Score	Rank
3-OH-C10-HSL	71353010	Y80, W84, Y88, D97, S155	-85.11	1	Y80, W84, Y88, D97, S155	-81.54	1
Quercetin aglycone	5280343	M135, S155	-52.95	2	M135, S155	-53.87	2
Quercetin 3-β-D-glucoside	5280804	Y88, S89	-45.81	3	**Y88**, S89, **N92**, **A94**	-53.40	3
Furanone C-30	10131246	Y80, T140, S155	-34.42	4	W84	-33.58	4


*Amino acid residues that bind only to the glucoside structure of quercetin 3-β-D-glucoside are in bold.*

**Table 5:** Results from molecular docking of structures of LasR protein of *P. aeruginosa* PAO1 with selected compounds.

Compound	Pubchem CID	Structures of LasR protein of *P. aeruginosa* PAO1														
		
		2UV0			6D6A			6D6L			6D6O			6D6P		
						
		Binding residue	Score	Rank	Binding residue	Score	Rank	Binding residue	Score	Rank	Binding residue	Score	Rank	Binding residue	Score	Rank
3-oxo-C12-HSL	3246941	Y56, T75, S129	-81.85	1	Y56, S129	-75.07	1	Y56, W60, D73	-76.40	2	Y56, S129	-76.80	2	T75, T115, S129	-78.90	2
Quercetin aglycone	5280343	T75, T115, L125, S129	-61.09	2	T75, Y93, L110, T115, S129	-70.07	3	R61, D65, T75, T115, S129	-70.37	3	R61, D65, T75, T115, S129	-69.56	3	R61, D65, T75, T115, S129	-67.13	3
Quercetin 3-β-D-glucoside	5280804	Y47, W60, Y64, T75, **V76**, T115, **L125**, S129	-47.80	3	**G38**, R61, T75, T115, **A127**, S129	-71.21	2	**Y47**, R61, **Y64**, D65, T75, T115, S129	-88.24	1	**Y47**, R61, **Y64**, D65, T115, S129	-84.24	1	**Y47**, R61, **Y64**, D65, T115, S129	-81.89	1
Furanone C-30	10131246	T75, T115, S129	-37.88	4	T75, T115, S129	-38.08	4	T75, T115, S129	-45.78	4	T75, T115, S129	-46.44	4	T75, T115, S129	-44.87	4


*Amino acid residues that bind only to the glucoside structure of quercetin 3-β-D-glucoside are in bold.*

## Discussion

Different cellular functions that affect food spoilage are influenced by signaling molecules accumulated as a function of QS. Consequently, many researchers have attempted to find alternatives that can inhibit this communication using natural sources that may reduce the virulence capacity of microorganisms. In the present study, we evaluated the effect of onion organic extracts and representative isolated compounds in QS model bacteria.

First, we identified the different phenolic compounds present in the organic extracts. The results showed that different types of glycosylated quercetin were found in both onion varieties. Studies have shown that flavonoids such as quercetin 4-*O*-glucoside and quercetin 3,4-*O*-diglucoside are the major compounds found in onion and compounds derived from kaempferol and isorhamnetin were identified as minor flavonoids (Slimestad et al., 2007; Lee et al., 2011; Pérez Gregorio et al., 2011). In addition, cyanidin 3-*O*-glucoside was the main anthocyanin present in red onion (Pérez Gregorio et al., 2011). Another study by Arabbi et al. (2004) has also shown that significant amounts of quercetin aglycone are found in concentrations of 48–56 mg/100 g on white onions and amounts of 38–94 mg/100 g in red onions. In addition, they reported that anthocyanin cyanidin was found in varieties of red onion contributing with 9.2% of total flavonoids (Arabbi et al., 2004).

The MIC of the extracts was equal to or greater than 125 μg/ml of dry extract for all the evaluated microorganisms. These results are related to those of Gopu et al. (2015), in which quercetin aglycone, one of the compounds found in the present study, showed a MIC value of 120 μg/ml for *C. violaceum* CV026. Another study by Al-Yousef et al. (2017) evaluated the ethyl acetate fraction of onion peel and its major compound quercetin 4-O-β-D-glucopyranoside as a possible QS inhibitor, finding a MIC value for *C. violaceum* ATCC 12472 of 500 μg/ml (Al-Yousef et al., 2017). Thus, it is possible that higher concentrations of onion extracts and quercetin are needed in order to fully inhibit the growth of the bacteria evaluated in the present study.

Quorum sensing regulates violacein production, a characteristic violet pigment produced by *C. violaceum*, which is induced by some types of *N*-acyl homoserine lactone molecules (Stauff and Bassler, 2011). Curiously, the strain used in the present work, *C. violaceum* ATCC 12472, is induced by *N*-(3-hydroxydecanoyl)-L-homoserine lactone, differing from the biosensor strain *C. violaceum* CV026 which is induced by *N*-hexanoyl-L-homoserine lactone (Morohoshi et al., 2008). We observed that neither WO-1 and WO-2 extracts nor quercetin 3-β-D-glucoside significantly inhibited violacein production, even though a tendency for an inhibitory effect can be observed (Figure 2). On the other hand, the RO-1 extract and quercetin aglycone significantly inhibited violacein production.

Several studies indicated violacein inhibition by different extracts. For instance, Oliveira et al. (2016) showed that the phenolic extract of *Rubus rosaefolius* (wild strawberry) reduced violacein production by up to 88%, especially in the concentration of 118.60 mg GAE/L, showing a higher inhibition than furanone, positive control for this experiment, which inhibited 68.6%. The same authors found that the enriched extract in phenolic compounds of *Malpighia emarginata* (*acerola*) significantly inhibited violacein production in all sub-MIC concentrations evaluated (Oliveira et al., 2017). Rodrigues et al. (2016) also showed that the phenolic extract of *Eugenia brasiliensis* (*grumixama*) presented a significant inhibition of violacein production in *C. violaceum* ATCC 6357. However, in these studies, no identification of which phenolic compounds specifically inhibited the phenotypes was performed. Therefore, the compounds that inhibited violacein production are likely different from those of the present study. In a work by Song et al. (2018), coral symbiotic bacteria were screened for their ability to inhibit violacein production in *C. violaceum* ATCC 12472, with 15% of the isolates presenting QS inhibition. Furthermore, the authors showed that rhodamine isothiocyanate which is produced by one of the isolates characterized as *Vibrio alginolyticus* was involved in the disruption of QS in *P. aeruginosa* PAO1.

For quercetin aglycone, the results showed that there was a significant inhibition of violacein production (*p* < 0.05). The results were comparable to those found by Gopu et al. (2015), who reported that in the presence of quercetin aglycone violacein production in *C. violaceum* CV026 was inhibited by up to 83.2% in a concentration of 80 μg/ml. We found inhibition in a concentration ranging from 15.6 to 125 μg/ml, even though we used a different strain of *C. violaceum* ATCC 12472. In contrast, quercetin 3-β-D-glucoside showed no significant inhibition of pigment production, possibly explaining the low anti-QS activity of the extracts that had glycosylated forms of quercetin as major compounds. This result indicates that the glycosylation of the molecule, or even other types of changes in the structure, could modify the antimicrobial and anti-QS activity of a phenolic compound. This hypothesis is supported by other studies reporting that changes in the flavone structure influence the biological activity of flavonoids (Xiao et al., 2014; Paczkowski et al., 2017; Xiao, 2017).

We have also performed molecular docking of quercetin aglycone and quercetin 3-β-D-glucoside with the QS transcription activator CviR protein of *C. violaceum* ATCC 12472 (Figure 5). The quercetin aglycone accommodates in the structure of the protein in a similar fashion to the autoinducer 3-OH-C10-HSL (Figure 5A,B), while the glycosylated quercetin presents an overall different molecular interaction with the protein, as observed by a larger portion of the bulky glycosylated quercetin molecule being exposed to the exterior of the structure (Figure 5C). In addition, the two quercetins bound at different amino acid residues, as well as the glucoside structure of quercetin 3-β-D-glucoside bound to other specific residues (Figure 5B,C). However, only quercetin aglycone and 3-OH-C10-HSL showed common binding site, suggesting that these compounds can compete to bind to the CviR protein of *C. violaceum* ATCC 12472 (Figure 5A,B).

The effect of the extracts on swarming motility was also evaluated. The expression of some virulence factors such as biofilm formation is associated with motility (Al-Yousef et al., 2017). Therefore, interferences in this phenotype can affect a microorganism’s pathogenicity. We observed for *P. aeruginosa* PAO1 a motility inhibition by RO-1 in all tested concentrations. On the other hand, *S. marcescens* MG1 swarming was inhibited by WO-2 only in the concentration of 125 μg/ml. Furanone C-30 was used as a positive control for swarming motility inhibition in the concentration of 100 μM and, as expected, presented the best phenotype inhibition, corroborating previous findings (Manefield et al., 2002; Hentzer et al., 2003). The other extracts did not present significant inhibition of the phenotype. These results are related to those obtained by Husain et al. (2015) in which the essential oil of *Mentha piperita* inhibited the swarming motility of *P. aeruginosa* PAO1. Vattem et al. (2007) also evaluated the effect of sub-lethal concentrations of phytochemicals of common fruits, herbs and spice extracts, demonstrating that they decreased *P. aeruginosa* PAO1 swarming motility by approximately 50% (Vattem et al., 2007). This behavior was also replicated in other bacteria, as in the study of Oliveira et al. (2016), which demonstrated that wild strawberry phenolic extract inhibited the swarming motility of a strain of *S. marcescens* and *A. hydrophila*, two bacteria found in refrigerated food products, besides inhibiting the production of prodigiosin, a red pigment found in *S. marcescens*, regulated by QS (Oliveira et al., 2016).

Our results also revealed that the two types of quercetin showed swarming inhibitory activity in *P. aeruginosa* PAO1 and *S. marcescens* MG1. Molecular docking of these quercetins with LasR protein of *P. aeruginosa* PAO1 revealed that they all could interact and accommodate in the pocket of the different structures of this protein (Figure 6B,C). The two quercetins and 3-oxo-C12-HSL, an autoinducer synthesized by *P. aeruginosa* PAO1, showed common binding sites (Figure 6A–C). In quercetin 3-β-D-glucoside, the flavonoid structure bound to these common amino acid residues, unlike its glucoside structure (Table 5). These results suggest that these quercetins could compete with autoinducer to bind to the LasR protein of *P. aeruginosa* PAO1. Our results confirm a previous docking study performed by Gopu et al. (2015) with quercetin aglycone and LasR protein. Biochemical studies such as those performed by Paczkowski et al. (2017) with purified LasR protein and different types of quercetin molecules should be performed in order to confirm these findings.

It is important to highlight that no study has evaluated in detail the effects of red and white onion extracts on QS regulated phenotypes. In a work by Rasmussen et al. (2005), libraries of plant extracts and isolated chemical compounds were created to evaluate which of these had anti-QS activity, using a selection system of QS inhibitors called QSIS. They evaluated extracts of spring and brown onion but there was no apparent QS inhibition in their assays. The answer to the absence of inhibition may be related to the extracts concentration, identity of the extracted compounds, extraction method and the systems used to detect the anti-QS activity. It could also point to the fact that their extract could be enriched in glycosylated phenolic compounds which we suggest have lower anti-QS activity.

Finally, we analyzed the effect of the extracts on biofilm production. Biofilms are known as microbial communities that adhere to surfaces and are protected by an adherent polymeric matrix (Hentzer et al., 2002). Studies have shown that QS communication plays an important role in the maturation process of these cellular aggregates (Hammer and Bassler, 2003; Morohoshi et al., 2007). Our experiments indicate that the extracts did not inhibit biofilm formation by *P. aeruginosa* PAO1 and *S. marcescens* MG1 at any given concentration (Figure 4A,C). However, experiments with quercetin and quercetin 3-β-D-glucoside showed conflicting results. For instance, the crystal violet assay suggested inhibition at concentration of 31.2 and 62.5 μg/ml of the glycosylated quercetin, and with the motility assay we also observed inhibition by both types of quercetins in the two evaluated microorganisms. But in the case of *P. aeruginosa* PAO1 there was no inhibition of biofilm at the concentration of 125 μg/ml (Figure 4A). We attempted to confirm these results by counting viable cells recovered from biofilms under these conditions, but no inhibition was observed at any of the tested concentrations (Figure 4B). Overall, these results suggest that neither the extracts, nor the quercetins evaluated in this study presented potential to inhibit biofilm formation at concentrations that supposedly inhibit QS. Therefore, we encourage the use of different methods in order to confirm a possible QS inhibiting candidate.

Generally, our results disagree with those of Al-Yousef et al. (2017). These authors evaluated the ethyl acetate fraction of onion peel and its major compound quercetin 4-*O*-β-D-glucopyranoside and found an inhibition of up to 64% in biofilm formation by *P. aeruginosa*. Interestingly, they used higher concentrations of extract and quercetin (up to 400 μg/ml) than in the present study where the highest concentration tested in QS inhibition experiments was 125 μg/ml. Additionally, it is important to note that in the Al-Yousef study, the major compound identified was quercetin 4-*O*-β-D-glucopyranoside, which in its structure has a sugar molecule attached to C4 of B ring in the flavone group, while the majority of glycosylated compounds present in the onion extracts evaluated in the present work have a sugar moiety attached to C3, taking into account that these structural changes would interfere with the effectiveness of the compound as an inhibitor.

Ouyang et al. (2016) evaluated the effect of quercetin aglycone on biofilm formation of *P. aeruginosa* PAO1 in sub-MIC concentrations. Their results showed that quercetin aglycone inhibited biofilm formation in concentrations ranging from 8 to 64 μg/ml. The concentration of 16 μg/ml had the best inhibitory effect (around 50% inhibition), similarly to azithromycin at 32 μg/ml, an antibiotic used in clinical treatments (Ouyang et al., 2016). Interestingly, when comparing their data to the results of the present study, inhibition was not consistent. For instance, concentrations of 32 and 64 μg/ml of quercetin presented less inhibition than 16 μg/ml. The reasons for these inconsistent results could be related to the hormesis effect (Calabrese, 2008), even though more studies are needed to confirm such hypothesis.

Another research conducted by Paczkowski et al. (2017) showed that different flavonoids, including quercetin, could specifically inhibit QS in *P. aeruginosa* PAO1. Structure-activity analyses demonstrated that the presence of two hydroxyl groups on the A ring of flavone, one at position 7 and at least one at any other position, are required for a potent inhibition of LasR/RhIR receptors. In addition, the authors have shown that rings B and C can accommodate many substitutions, with exception of methyl groups in B ring, which are not tolerated, because they are too bulky. Biochemical analysis revealed that flavonoids function in a non-competitive way to prevent binding of QS receptors to DNA, altering the transcription of QS-controlled target promoters and suppressing the production of virulence factors (Paczkowski et al., 2017). However, the study evaluated non-glycosylated compounds; therefore, the question whether these modifications would work for compounds that have sugar substitutions attached to any of the flavonoid rings still remains.

Overall, quercetin aglycone had better inhibitory activity over QS in *C. violaceum* ATCC 12472 than quercetin 3-β-D-glucoside. On the other hand, the two quercetins inhibited motility of *P. aeruginosa* PAO1 and *S. marcescens* MG1. The results of the biological and *in silico* analyses confirmed that the structure of the compounds interferes with anti-QS activity indicating that the low activities of organic extracts of onion varieties may be related to the glycosylation of phenolic compounds. In addition, the response of the tested bacteria may be different as a function of the amino acid variations and structures of the receptor QS proteins. It would be interesting to test different phenolic compounds with or without modifications and in different bacteria to strengthen these findings. Xiao et al. (2014) and Xiao (2017) demonstrated that glycosylation generally reduces the bioactivity of flavonoids. This phenomenon has been observed for different properties including antioxidant, anti-inflammatory, antibacterial and antifungal activities.

## Conclusion

The effect of organic extracts of red and white onion and their major constituents on QS controlled phenotypes has been evaluated. Different glycosylated quercetins were found in both onion varieties. The onion organic extracts showed inhibition of violacein production and swarming motility. Violacein production was significantly inhibited by quercetin aglycone, while glycosylated and quercetin aglycone inhibited motility of *P. aeruginosa* PAO1 and *S. marcescens* MG1. *In silico* studies suggested a better interaction and accommodation of quercetin aglycone in the structures of the CviR protein of *C. violaceum* ATCC 12472 than the glycosylated compound. On the other hand, the two quercetins were able to bind and accommodate in the pocket of LasR protein of *P. aeruginosa* PAO1. Surprisingly, biofilm formation was not affected by any extracts or the quercetins tested in this study. These results suggest that the extracts and isolated compounds of onions could interfere in the antimicrobial and anti-QS activity, but interference on QS was limited to violacein production and swarming motility.

## Author Contributions

All authors listed have made a substantial, direct and intellectual contribution to the work, and approved it for publication.

## Conflict of Interest Statement

The authors declare that the research was conducted in the absence of any commercial or financial relationships that could be construed as a potential conflict of interest.

## Footnotes

1 http://www.rcsb.org/pdb/home/home.do

2 https://www.qiagenbioinformatics.com/

## Supplementary Material

FIGURE S1 LC(ESI)-MS/MS spectra of chromatographic peaks obtained from red and white onions. **(A)** Peak 1 – Quercetin 3,4-*O*-diglucoside; **(B)** peak 2 – Isorhamnetin 3,4′-diglucoside; **(C)** peak 3 – Quercetin 3-*O*-glucoside; **(D)** peak 4 – Quercetin 4′-*O*-glucoside; **(E)** peak 5 – Isorhamnetin4-glucoside; **(F)** peak 6 –Quercetin glycoside; **(G)** peak 7 – Quercetin aglycone, and **(H)** peak 8 – Cyanidin 3-*O*-glucoside in positive ion mode.
