# Supplementary figures and images for: Effect of Quercetin Rich Onion Extracts on Bacterial Quorum Sensing
Source: Front Microbiol. 2019 Apr 24;10:867. doi: 10.3389/fmicb.2019.00867 (PMC6492534; doi:10.3389/fmicb.2019.00867)

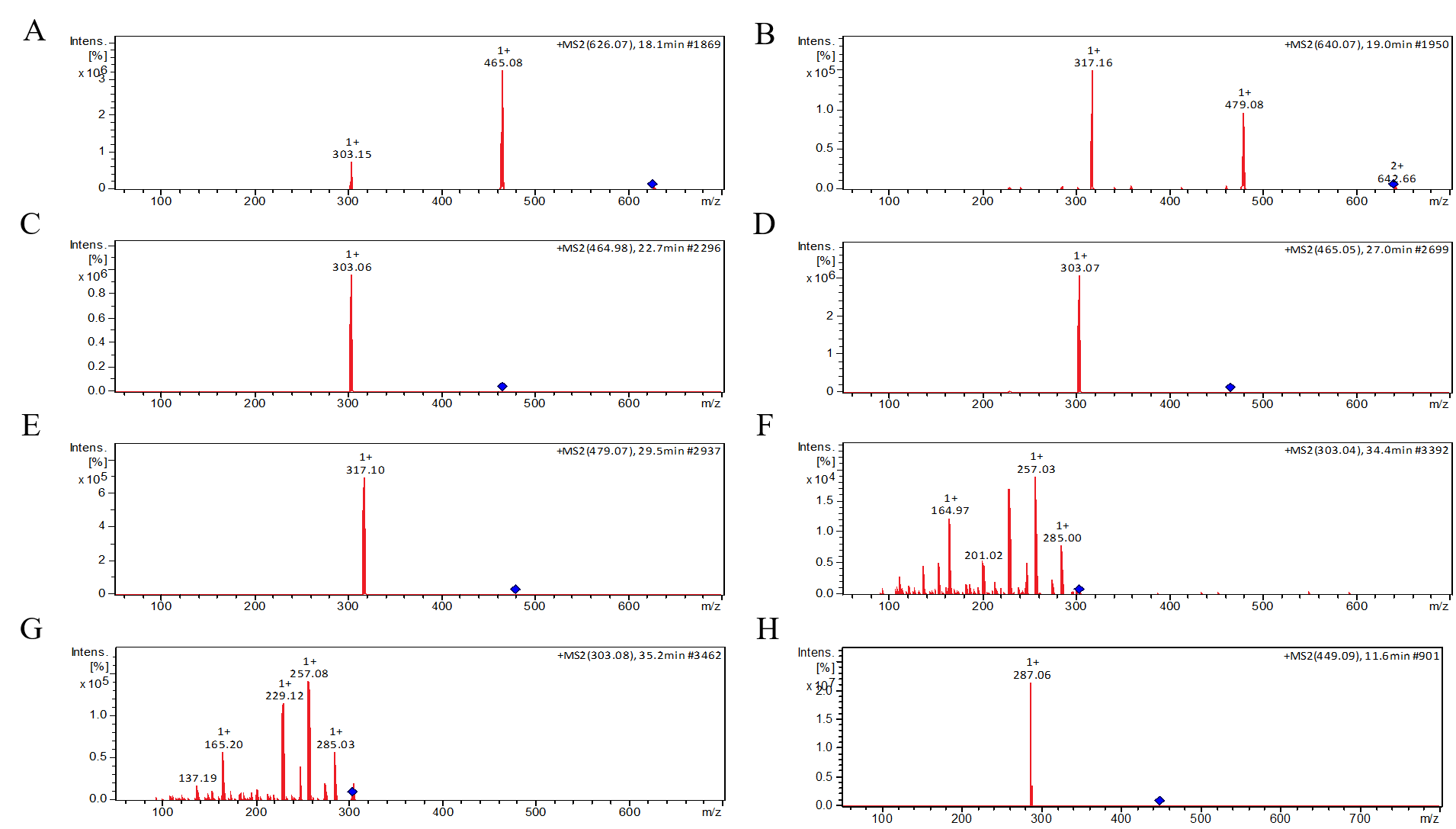

Supplement: FIGURE S1 — LC(ESI)-MS/MS spectra of chromatographic peaks obtained from red and white onions. (A) Peak 1 – Quercetin 3,4-O-diglucoside; (B) peak 2 – Isorhamnetin 3,4′-diglucoside; (C) peak 3 – Quercetin 3-O-glucoside; (D) peak 4 – Quercetin 4′-O-glucoside; (E) peak 5 – Isorhamnetin4-glucoside; (F) peak 6 –Quercetin glycoside; (G) peak 7 – Quercetin aglycone, and (H) peak 8 – Cyanidin 3-O-glucoside in positive ion mode. [file Image_1.TIF]
